# CHD1 Remodels Chromatin and Influences Transient DNA Methylation at the Clock Gene *frequency*

**DOI:** 10.1371/journal.pgen.1002166

**Published:** 2011-07-21

**Authors:** William J. Belden, Zachary A. Lewis, Eric U. Selker, Jennifer J. Loros, Jay C. Dunlap

**Affiliations:** 1 Department of Genetics, Dartmouth Medical School, Hanover, New Hampshire, United States of America; 2 Department of Biochemistry and Microbiology, Rutgers, The State University of New Jersey, New Brunswick, New Jersey, United States of America; 3 Institute of Molecular Biology, University of Oregon, Eugene, Oregon, United States of America; 4 Department of Biochemistry, Dartmouth Medical School, Hanover, New Hampshire, United States of America; University of Texas Southwestern Medical Center and Howard Hughes Medical Institute, United States of America

## Abstract

Circadian-regulated gene expression is predominantly controlled by a transcriptional negative feedback loop, and it is evident that chromatin modifications and chromatin remodeling are integral to this process in eukaryotes. We previously determined that multiple ATP–dependent chromatin-remodeling enzymes function at *frequency* (*frq*). In this report, we demonstrate that the *Neurospora* homologue of *chd1* is required for normal remodeling of chromatin at *frq* and is required for normal *frq* expression and sustained rhythmicity. Surprisingly, our studies of CHD1 also revealed that DNA sequences within the *frq* promoter are methylated, and deletion of *chd1* results in expansion of this methylated domain. DNA methylation of the *frq* locus is altered in strains bearing mutations in a variety of circadian clock genes, including *frq*, *frh*, *wc-1*, and the gene encoding the *frq* antisense transcript (*qrf*). Furthermore, *frq* methylation depends on the DNA methyltransferase, DIM-2. Phenotypic characterization of *Δdim-2* strains revealed an approximate WT period length and a phase advance of approximately 2 hours, indicating that methylation plays only an ancillary role in clock-regulated gene expression. This suggests that DNA methylation, like the antisense transcript, is necessary to establish proper clock phasing but does not control overt rhythmicity. These data demonstrate that the epigenetic state of clock genes is dependent on normal regulation of clock components.

## Author Summary

Circadian rhythms facilitate daily changes in gene expression via a transcriptional negative feedback loop. In eukaryotes, chromatin remodeling is an integral part of transcriptional regulation and is proving to be one of the major determinants for the proper timing and amplitude of clock-gene expression. We describe here the action of chromodomain helicase DNA–binding (CHD1), one of two ATP–dependent chromatin-remodeling enzymes required for normal circadian regulated gene expression of the central clock gene *frequency* (*frq*). Molecular analysis of strains lacking *chd1* indicates that CHD1 is required for remodeling chromatin structure at the *frq* locus as a part of the daily clock cycle. Moreover, we discovered DNA methylation in the promoter of *frq* that diminishes over time in the absence of light/dark cycles and determined that normal DNA methylation appears to require a functional clock. The DNA methyltransferase DIM-2 is responsible for this DNA methylation, and the DNA methylation is required for proper phasing of clock gene expression. Collectively, these data demonstrate a close connection among chromatin remodeling, DNA methylation, and clock gene expression.

## Introduction

Virtually all eukaryotes have the ability to control temporal gene expression as a function of time-of-day. The molecular mechanism of circadian rhythms is conserved among animals and fungi and involves a transcriptional negative feedback loop [1]–[5]. In *Neurospora*, the positive elements are the White Collar (WC) proteins, which drive rhythmic expression of the negative element *frq*. WC-1 and WC-2 are GATA-type zinc finger transcription factors that heterodimerize via PAS (Per-Arnt-Sim) domains to form the White Collar Complex (WCC) [6]–[9]. Rhythmic and light-activated WCC-driven expression of *frq* is regulated by two cis-acting sequences in the promoter, the Clock Box (C-box) and the proximal light regulated element (PLRE) [10], [11].

In the negative arm of the loop, translated FRQ protein associates with the FRQ-interacting RNA helicase, FRH [12], and undergoes regulated nuclear entry [13] modulated in part by the phosphorylation state of FRQ [14]. FRQ-FRH is believed to inhibit *frq* expression by promoting inactivation of the WCC through phosphorylation [15], [16] and via direct binding to the WCC [17]. In addition, circadian regulated *frq* expression is partially controlled by a chromatin-remodeling event that promotes accessibility to the C-box promoter element [18]. Once FRQ is synthesized, it undergoes progressive phosphorylation [19]–[21] while feeding back to inhibit its expression [22] and it is eventually turned over in a reaction that involves an F-box/WD40 protein FWD [23] and the SKP-cullin complex [24], thus releasing its negative effects on transcription. A plethora of data indicates FRQ phosphorylation and turnover establish period but less is known about the molecular mechanisms underlying phase determination.

Rhythmic changes in chromatin structure have been correlated with changes in transcriptional activity of clock-associated loci. In mammalian cells, rhythmic modifications include acetylation of histone H3 at *Per1*, *Per2* and *Cry*, and acetylation of H4 at *Per1*, with the peak in acetylation occurring during the transcriptional activation phase [25], [26]. In contrast, during the repressive phase, histone H3 associated with *Per1* and *Per2* becomes methylated at K27 [27]. There are also rhythmic changes at the clock-controlled gene (*ccg*) encoding the D-element binding protein (*Dbp*), including H3K9me2 and HP1 binding, suggesting that at least one *ccg* is regulated by facultative heterochromatin formation [28]. In addition, the role of CLOCK as a histone acetyltransferase [29], and the acetylation of BMAL1 [30], combined with the observation that the NAD^+^-dependent histone deacetylase SIRT1 modulates the amplitude of circadian regulated gene expression [31], [32] has garnished attention. More recently, the mixed-lineage leukemia (MLL1) lysine methyltransferase (KMT2A) has been show to associate with CLOCK-BMAL1 and is required for rhythmic expression of clock genes [33]. Additionally, the histone demethylase JMJD5 (KDM8) is rhythmically expressed and is required for normal rhythms in both the *Arabidopsis* and mammalian clock suggesting that oscillations in H3K36 methylation are also an important component of clock function [34]. Current models of circadian gene regulation suggest that the clock controls its own phase-specific chromatin modifications, which help confer levels of expression appropriate for the time-of-day. How these “marks” are established after entrainment, transferred after replication, and ultimately interpreted so that rhythms are maintained and asynchronous expression is prevented are active areas of research.

It is clear that chromatin structure is instrumental for appropriate circadian regulation of transcription. In fact, circadian changes in nucleosome location are known to occur at both *frq* and *mDbp* [18], [35], indicating that the clock controls chromatin architecture at a subset of clock-regulated loci. Consistent with this idea, circadian regulated remodeling at *frq* involves the ATP-dependent chromatin-remodeling enzyme CLOCKSWITCH (CSW-1) [18]. Moreover, *Drosophila* KISMET protein, a member of the CHD subfamily of ATP-dependent remodeling enzymes, was shown to be a key regulator of photoresponses [36]. RNAi knockdowns of the *kis* gene resulted in activity rhythms in constant light similar to the *cry^b^* mutation [36].

In this report, we show that an additional remodeling enzyme CHD1, remodels chromatin at *frq*, is necessary for normal *frq* expression, and is involved in regulating chromatin structure at *frq*. Surprisingly, we report that DNA in the *frq* promoter is methylated and we observe an expanded domain of DNA methylation at *frq* and other light/clock regulated genes in *Δchd1*. This was unexpected because previously, only repeated DNA sequences were found to be methylated and presumably silenced (reviewed in [37]). We show transient and reversible methylation that is associated with regulatory and coding sequences of genes that are actively expressed. This normal DNA methylation at *frq* is altered in strains bearing mutations in circadian clock genes and the *frq* antisense transcript *qrf*, and it is catalyzed by the DNA methyltransferase DIM-2. Strains lacking *dim-2* exhibit a small phase advance, suggesting that the methylation serves to limit the onset of circadian regulated transcription. These results demonstrate that transient, facultative, and presumably regulated DNA methylation also occurs in fungi and that chromatin remodeling and DNA methylation combine to fine turn circadian regulated gene expression.

## Results

### *CHD1* is required for normal *frq* expression

Previously, we generated and screened 19 *Neurospora* knockout strains in which proposed ATP-dependent chromatin-remodeling enzymes were deleted, to identify genes necessary for remodeling chromatin structure at *frq* [18]. As is typical for circadian research in *Neurospora*, these knockouts were generated in a *ras-1^bd^* genetic background in order to facilitate subsequent screening of the overt rhythm in conidiation [38], [39]. In this genetic background, we found that two remodeling enzymes (orthologues of Sth1 and Chd1) appeared essential for viability and a third, CSW-1, whose closest homologues are Fun30 in *Saccharomyces cerevisiae*, ETL1 in mice, and SMARCAD in humans, remodels chromatin at *frq* [18]. Subsequent analysis indicated that *Δchd1* is synthetically lethal with *ras-1^bd^*, explaining our inability to isolate viable ascospores that contain both *Δchd1* and *ras-1^bd^*. We have since obtained a viable homokaryotic knockout of the *chd1* homologue, NCU03060 in a WT background, from the *Neurospora* Genome Project knockout consortium [40]. Verification of the *Δchd1* knockout strain and characterization of its associated phenotypes can be found in Figure S1.

**Figure 1:**
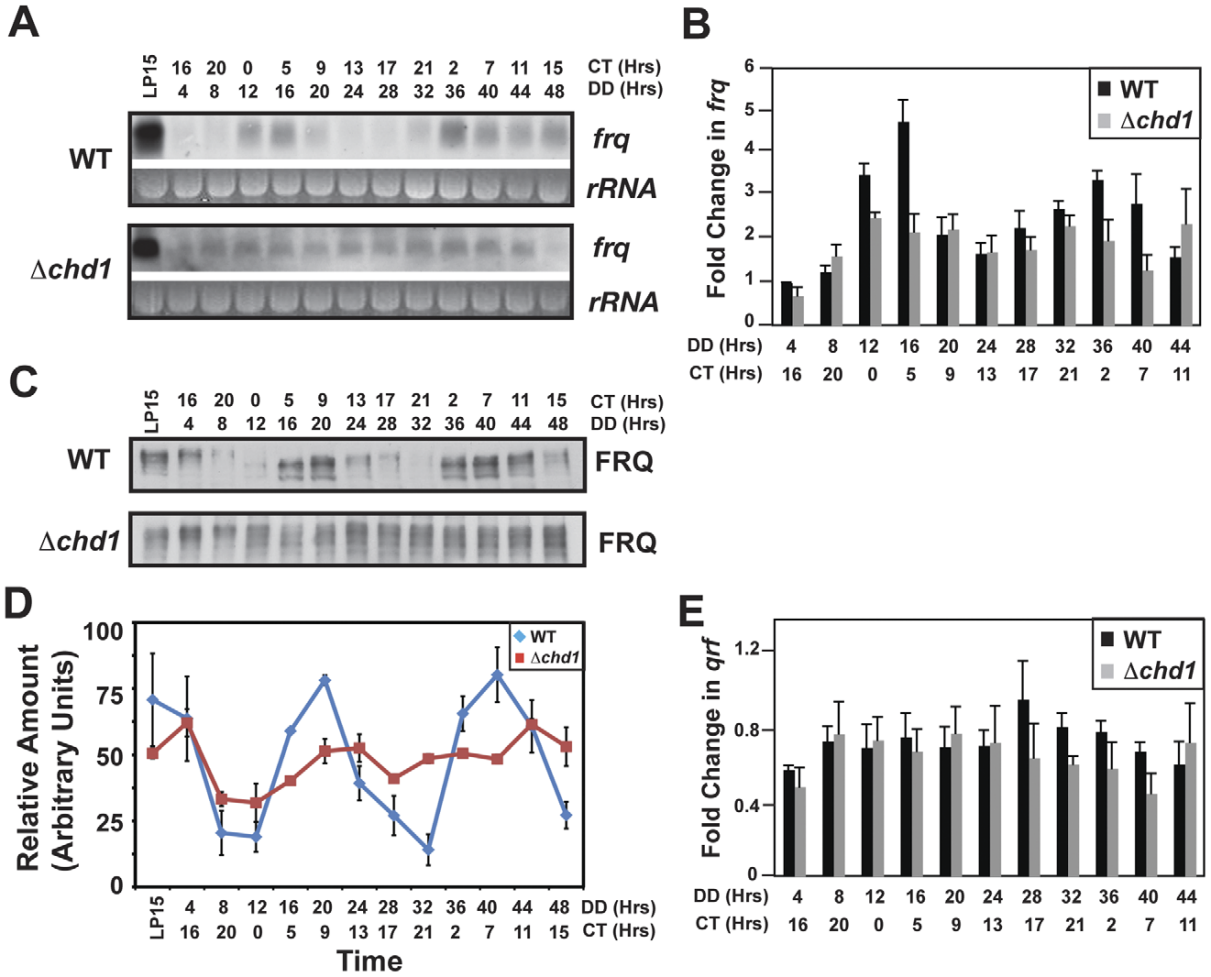
CHD1 is required for proper *frq* regulation. (A) Northern blot and (B) Real-time RT-PCR analysis of *frq* RNA levels in *Δchd1* and WT strains. (C) Immunoblot analysis on cell lysates using a FRQ antibody to compare expression in WT versus *Δchd1* [19]. These and comparable data from three replicate experiments were quantified and are plotted in panel D which shows rapid loss of rhythmicity. (E) Real-Time RT-PCR using a *qrf*-specific primer in an RT reaction followed by quantitative PCR of the *qrf* transcript. DD indicates the length of time that samples were exposed to dark conditions and the corresponding circadian time (CT) is also shown. In this and subsequent figures LP15 identifies cultures that were kept in darkness for 24 hours and then exposed to saturating light treatment for 15 minutes prior to harvesting. Error bars represent the standard error.

Because *chd1* is essential when combined with *ras-1^bd^*, we were unable to assay clock phenotypes with race tubes, the standard assay used to measure circadian regulated conidiation. Therefore, circadian rhythmicity was analyzed molecularly after a standard light to dark transfer by examining the normally rhythmic expression of *frq* mRNA transcript and protein. Northern blot analysis (Figure 1A) and quantitative RT PCR (Figure 1B) revealed that in the *Δchd1* strain, *frq* expression was diminished and the amplitude of rhythmic RNA accumulation was significantly reduced and rapidly graded to arhythmicity. Western blot analysis (Figure 1C) confirmed that clock function was disrupted in the *Δchd1* strain. FRQ was detected as multiple phosphorylated forms, indicating newly synthesized FRQ was continually replacing protein destined for turnover. Quantification of the western blot is shown in Figure 1D and is consistent with the RNA expression data showing that CHD1 is required for sustained rhythmicity. We also observed a striking defect in chromatin remodeling at the antisense promoter (see below) so we examined expression of the antisense transcript by quantitative RT-PCR using a primer specific for antisense in the reverse transcriptase reaction. We found no significant differences between WT and *Δchd1* strains (Figure 1E). Although we did not see a rhythm in *qrf* in a true wild-type strain, we did confirm the low amplitude rhythm in the *ras-1^bd^* strain previously reported [41] (data not shown).

**Figure 2:**
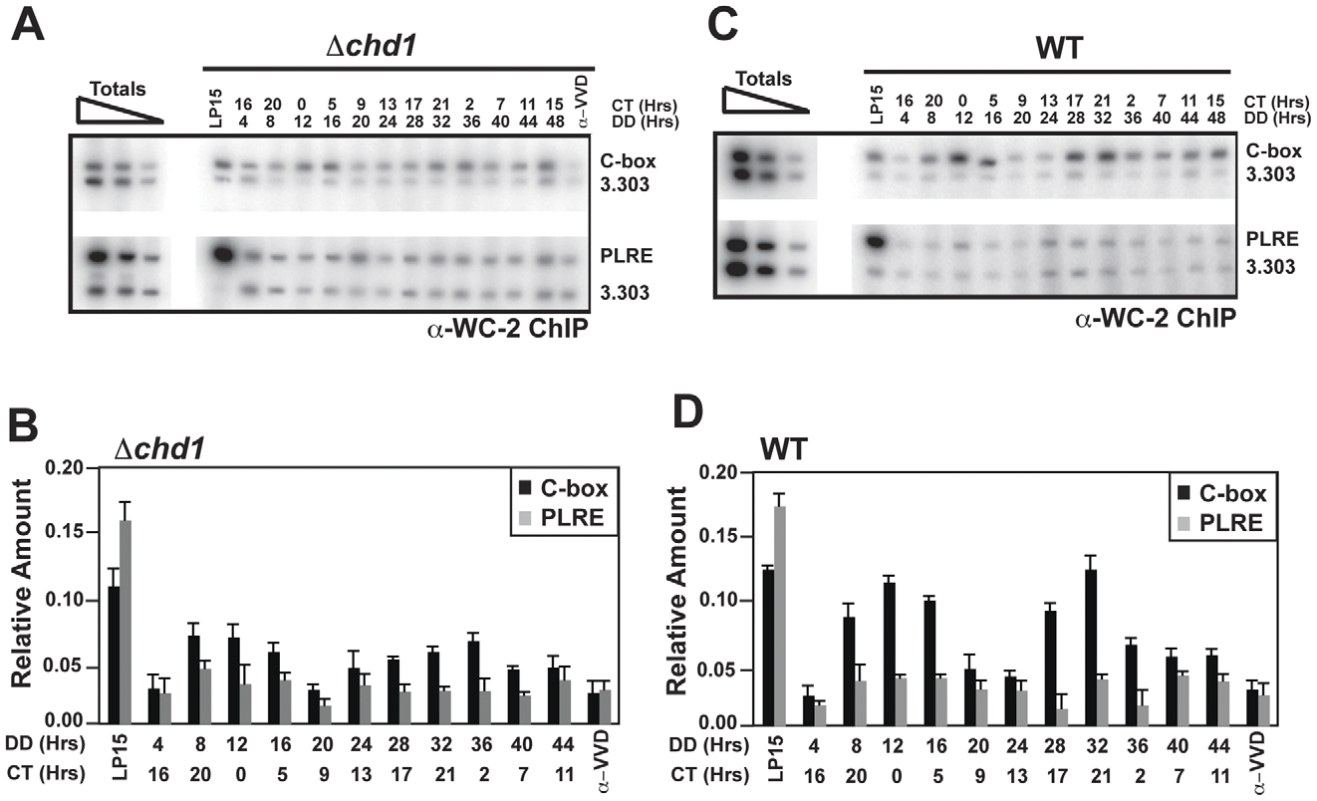
Binding of WC-2 to the *frq* promoter in *Δchd1*. ChIP analyses examining WC-2 binding at the C-box and PLRE in *Δchd1* (A and B) and WT (C and D) strains. The panels show a dilution series of the total lysate and the 3.303 oligo pair is used as a measure of nonspecific background [18]. Error bars represent the standard error. The totals consisted of a 1∶100, 1∶1,000 and 1∶10,000 dilution of the starting DNA present in the cross-linked lysates.

To further define *frq* expression, we used chromatin immunoprecipitation (ChIP) to assay the association of WC-2 with the *frq* promoter in *Δchd1* (Figure 2A and 2B). We consistently detected a subtle, low-amplitude rhythm in WC-2 association with the C-box, but binding was reduced approximately 50% when compared with WT, consistent with the observed reduction in *frq* mRNA (Figure 2C and 2D). The low amplitude rhythms in *frq* message and rhythmic WC-2 binding to the C-box suggest a more supportive rather than immediately essential role for CHD1 in clock function; however, these analyses only monitor clock function for two circadian cycles.

To confirm the effects of *Δchd1* on clock function and to examine *frq* expression over an extended circadian time course, we used an indirect method to measure rhythmicity of the core oscillator in real time using a *frq promoter-luciferase* reporter construct [42]. WT and *Δchd1* strains containing the pVG110 construct integrated at the *his-3* locus were grown on mini-race tubes and LUC activity was initially monitored over the entire tube and at region near the inoculation point (Figure S2). While clear robust rhythms were observed in WT, the *Δchd1* strain typically displayed a single peak of *frq*-driven LUC activity with no evidence of sustained circadian regulation. Together, these data demonstrate that CHD1 is required to maintain normal high amplitude rhythms of WC-2 binding and *frq* mRNA accumulation, and is essential for persistent circadian expression of *frq* in synchronized *Neurospora* cultures.

### CHD1 remodels chromatin at *frq*

**Figure 3:**
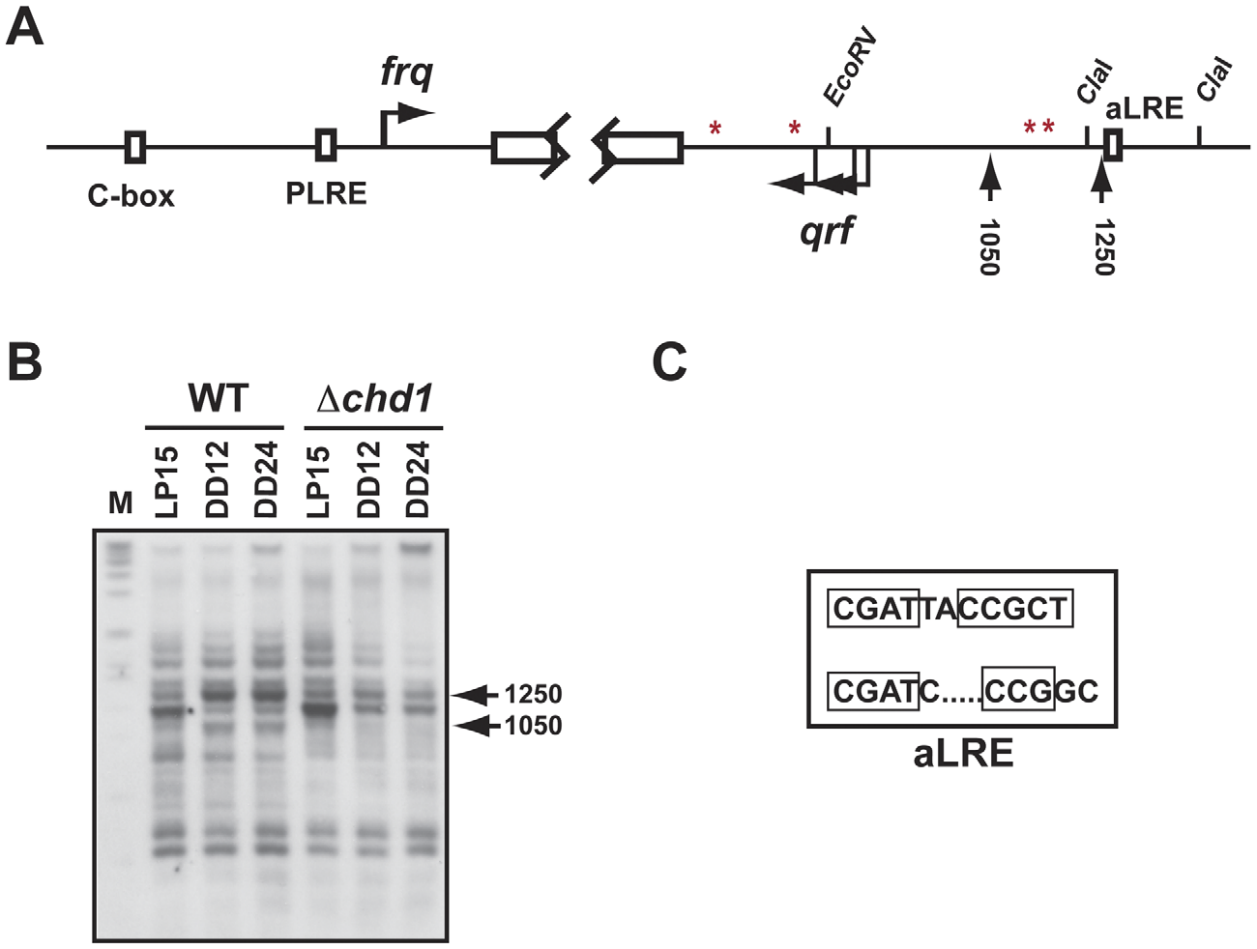
CHD1 is needed for remodeling at *frq*. (A) Schematic representation of the *frq* locus with vertical arrows (1050 site and 1250 site) indicating the regions where remodeling is observed. Horizontal arrows indicate the major start sites of transcription for both *frq* and *qrf*. Red asterisks indicate the poly A sites for *frq*. (B) Chromatin structure was visualized by indirect end-labeling of DNA after partial MNaseI digestion. Nuclei were isolated from WT and *Δchd1* strains grown in the dark (DD12 and DD24) and after a 15-minute light-pulse (LP15). The two arrows (1050 and 1250) identify specific MNase I sites in the region of the *qrf* promoter. (C) A consensus WCC binding site called the antisense light response element (aLRE) is located in the *qrf* promoter.

Previously, we showed that chromatin in the *frq* promoter region, near the transcriptional start site (TSS) and the PLRE, is remodeled in response to light treatment but that this remodeling is independent of CSW-1 [18]. We suspected that this light-dependent remodeling might be catalyzed by CHD1 because of the failure to properly express *frq* in *Δchd1* strains. Therefore, we asked if CHD1 is responsible for catalyzing the opening and closing of chromatin structure by comparing *Δchd1* and WT nuclei using a limited MNase I digestion. Nuclei were isolated from 48-hour old cultures grown in the dark for 12 hours (CT0) and 24 hours (CT12), and treated with a 15 minute light-pulse (LP15) given after 24 hrs in darkness. Regions in *frq* were examined by partial MNaseI digestion followed by indirect end-labeling (Figure 3A). Within the region surrounding the PLRE and TSS, we consistently only observed a very minor remodeling defect in *Δchd1* compared to WT (Figure S3). We extended our analysis to regions near the C-box promoter element, a region shown to be remodeled by CSW-1 [18], and did not observe any CHD1-dependent remodeling in that region (data not shown). In contrast to the subtle effect seen in the sense promoter, we detected unequivocal CHD1-dependent changes in chromatin structure in the antisense promoter (Figure 3B). A sequence near the 1250 site in the *qrf* promoter closely matches the imperfect repeat consensus sequence of the C-box and PLRE previously described [10]. WC-2 binding to the antisense light response element (aLRE) has been observed in WC-2 ChIP-seq experiments [43]. As indicated above, chromatin remodeling in the *frq* promoter persists in *Δchd1* and may account for the low amplitude rhythms in *frq* mRNA and WC-2 binding observed in *Δchd1* mutants (Figure 1A and Figure 2A). This observation is consistent with previous work and suggests that multiple chromatin remodeling enzymes regulate *frq* including the previously identified CSW-1 protein [18]. These data cumulatively indicate that chromatin structure at *frq* in a *Δchd1* strain is different from that in WT and implicates CHD1 in maintaining chromatin architecture at the *frq* locus.

### DNA methylation occurs at the *frq* promoter

**Figure 4:**
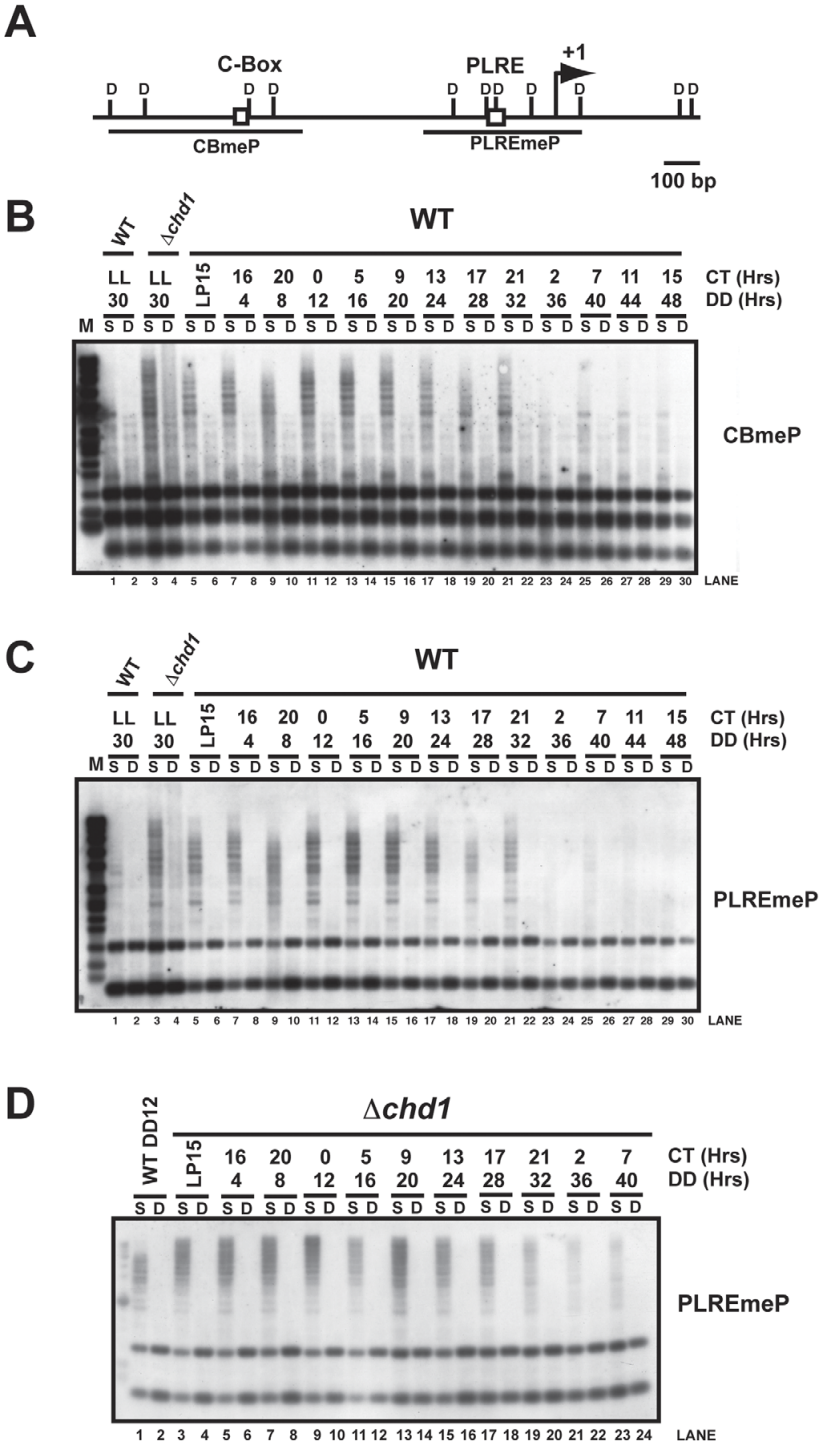
DNA methylation occurs at *frq*. (A) Schematic diagram of the *frq* locus illustrating the location of the probes. (B) Genomic DNA was isolated from WT and *Δchd1* strains grown in constant light (LL) at 30°C (Lanes 1–4) and compared to that from a WT strain grown in constant darkness (DD) for the indicated times over a 48-hrs time course. DNA was digested with *Sau*3AI (S) and *Dpn*II (D) and probed with the C-box probe. (C) The blot represented in B was probed for the PLRE. (D) DNA was isolated from *Δchd1* grown under standard circadian entrainment, handled as described and examined using the PLRE to detect DNA methylation.

Curiously, in the Southern blot data associated with the MNase I remodeling assay described in Figure S3, we routinely observed that a large percentage of the DNA isolated from *Δchd1* was resistant to digestion with the restriction endonuclease *Nco*I. *Nco*I is sensitive to cytosine methylation, which suggested the possibility that *frq* DNA from *Δchd1* is hypermethylated. Tests using other methylation-sensitive enzymes strongly supported this notion (Figure 4A–4D, and data not shown). This apparent DNA methylation at *frq* is surprising; in *Neurospora*, about 2% of cytosines are methylated, but in a variety of strains and under all conditions previously tested methylation has been shown to exist almost exclusively in relics of RIP (repeat-induced point mutation) and rDNA [37], [44], [45].

A common assay for examining DNA methylation in *Neurospora* utilizes the methylation sensitive restriction endonuclease *Sau3*AI and its methylation insensitive isoschizomer, *Dpn*II, followed by Southern blot analysis [46]. We first examined DNA methylation in WT and *Δchd1* strains grown in constant light for 48 hrs at 30°C, which results in constitutive, non-rhythmic *frq* expression, and compared this with DNA methylation observed in WT and *Δchd1* strains grown in constant darkness for two circadian cycles. Cultures were synchronized by a transfer from constant light at 25°C to constant darkness at 25°C and samples were collected every four hours (Figure 4). Southern blots were probed with DNA corresponding to the C-box (CBmeP) and PLRE (PLREmeP) to check for methylation at these sites (Figure 4A). The appearance of partially digested DNA in the *Sau3*AI digests suggested that the *frq* promoter is methylated (Figure 4B and 4C) under these conditions of growth in light followed by dark, and *frq* is hypermethylated in the *Δchd1* strain relative to WT grown under the same conditions (Figure 4B and 4C, lanes 1–4). To ensure that inhibition of *Sau3*AI digestion at *frq* was due to DNA methylation, we routinely stripped and reprobed blots for a region of the mitochondrial genome (Figure S4 and data not shown). Mitochondrial DNA, which is not methylated, but is at much higher concentrations relative to nuclear DNA, was completely digested when compared with *frq* at similar time points (Figure S4). Interestingly, the DNA methylation at the *frq* promoter decreased with time in darkness, although not in an overtly circadian manner (Figure 4B and 4C, lanes 5–30). In addition, promoter methylation appeared greatly reduced in cultures grown in constant light for extended periods (48 hours in light). In a *Δchd1* strain over the circadian time course *frq* promoter hypermethylation was seen at all time points examined (Figure 4D, compare lanes 1 and 2 with lanes 3–24).

**Figure 5:**
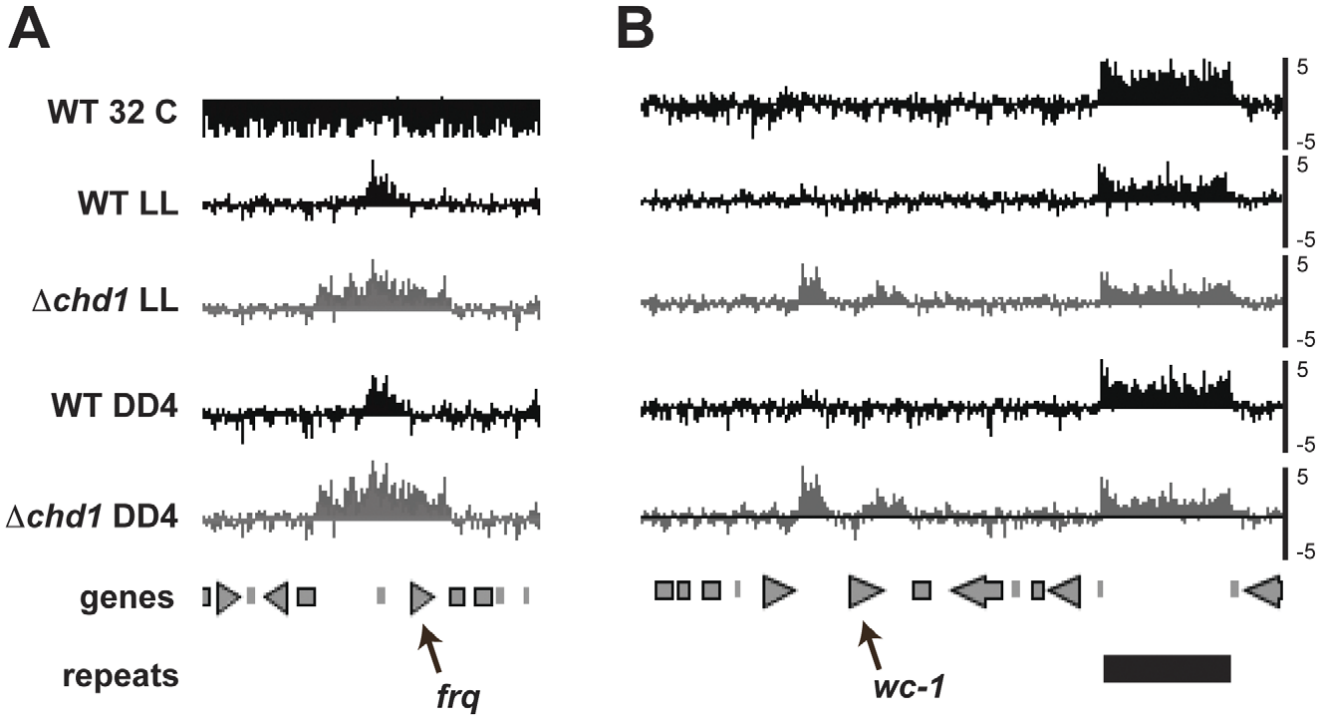
DNA methylation at *frq* and *wc-1*. Enrichment values for MeDIP experiments are shown at *frq* and *wc-1* loci as log2 values (right). MeDIP data for WT and *Δchd1* strains grown in constant light (LL) or for four hours in the dark (DD4) are shown below a plot of previously published MeDIP data obtained from WT grown at 32 degrees (WT 32) [45]. The positions of predicted genes are shown in grey and an annotated repeat near *wc-1* is shown in black; *frq* and *wc-1* are indicated.

The unanticipated observation of DNA methylation at *frq* immediately suggested the possibility that other genetic loci were similarly modified and similarly affected by loss of *chd1*. To examine this we performed methylated DNA immunoprecipitation (MeDIP), followed by hybridization of differentially labeled input and MeDIP fractions to a high-density chromosome VII (LGVII) microarray (MeDIP-chip) [45]. MeDIP-chip experiments using genomic DNA from WT or *Δchd1* cultures grown in constant light or in the dark for four hours revealed increased DNA methylation in the *Δchd1* strain, consistent with Southern blot analyses. A peak of DNA methylation at the *frq* promoter was observed in the WT strain. In the *Δchd1* strain, we observed an expanded domain of DNA methylation, which covered the entire *frq* promoter and open reading frame (Figure 5A). We next examined DNA methylation across the entire chromosome and found a peak of DNA methylation in the promoter of *wc-1* in *Δchd1* that was absent in the WT strain (Figure 5B). DNA methylation at *wc-1* in *Δchd1* was confirmed by a methyl-sensitive Southern blot, further supporting the validity of the MeDIP-Chip data (Figure S6). In contrast to *frq* and *wc-1* where methylation is influenced by loss of CHD1, DNA methylation at RIP'd regions was similar in WT and *Δchd1* (Figure 5B and data not shown), suggesting that CHD1 does not affect DNA methylation at constitutive heterochromatin. Because previous studies clearly showed a lack of methylation at the *frq* locus, it seems likely that particular growth conditions may be important to observe methylation and may indicate that it is dynamic [45]. Incidentally, when growth conditions consisting of elevated temperatures and extended light similar to previous work was used in this study, we failed to see significant methylation at *frq* (Figure 4B and 4C, lanes 1 and 2) supporting the notion that light-dark transitions or perhaps circadian entrainment are needed for methylation at *frq*.

We extended our analysis of the breadth of DNA methylation by examining more parts of the *frq* locus as well as other genes (Figure S5). Consistent with Figure 5, Southern hybridization confirmed that DNA methylation within the *frq* coding region is only seen in *Δchd1*, not WT. A third light and clock regulated gene, *vvd* [47] is similarly seen to be methylated in *Δchd1* but not in WT (Figure S5E and S5F) but another light and clock controlled gene *eas* (*ccg-2*) appeared never to be methylated, even in *Δchd1* (Figure S5C and S5D). Lastly, we examined the well-studied methylated Ψ_63_ region and found no discernable difference between WT and *Δchd1* (Figure S5G and S5H), consistent with the microarray data, which revealed that CHD1 does not effect the strong methylation seen at relics of RIP.

### Components of the clock impact normal methylation patterns

**Figure 6:**
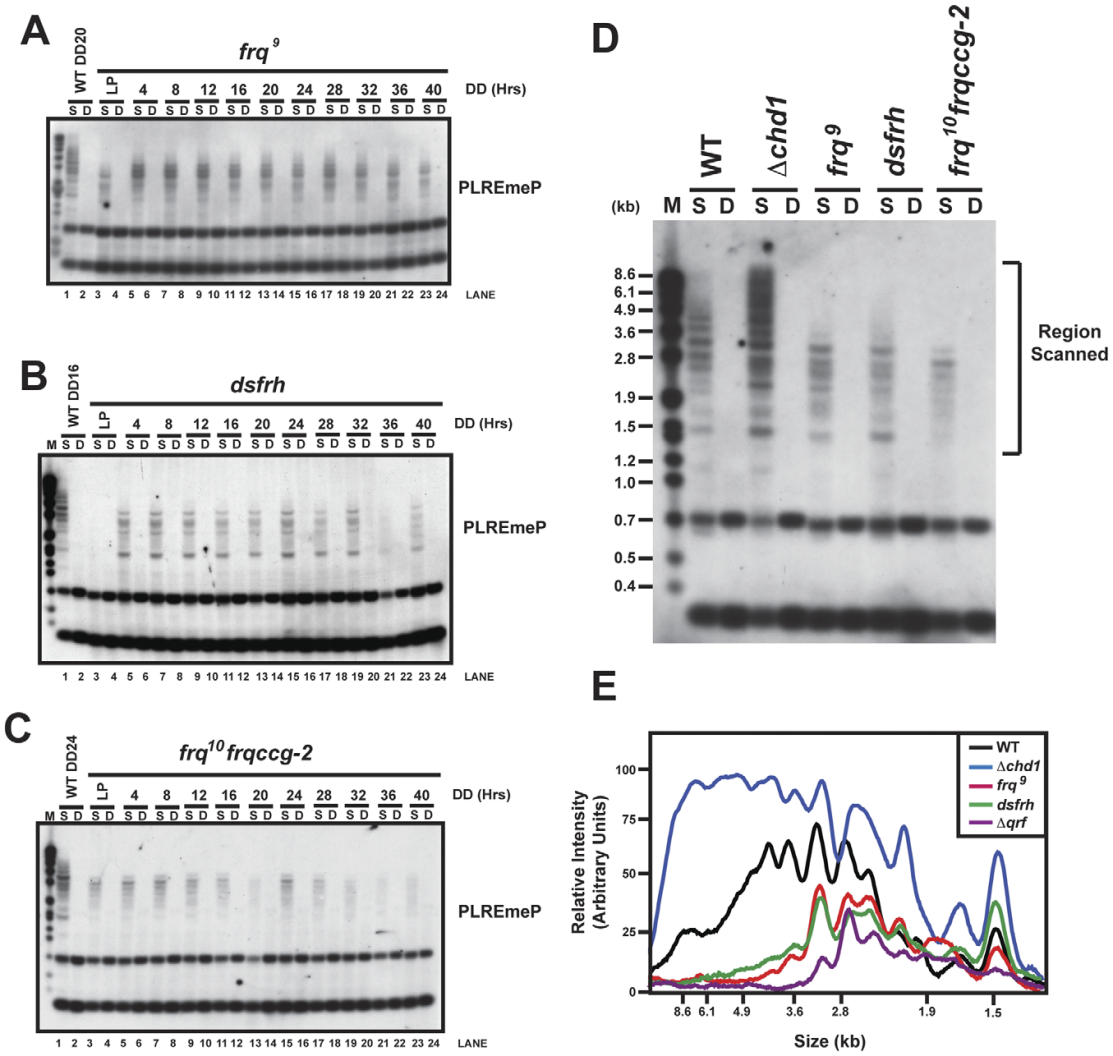
FRQ and the antisense transcript are required for normal DNA methylation. (A)The methylation status of the *frq* promoter was examined in *frq^9^*, (B) a FRH knock-down strain *dsfrh*, and (C) a strain with reduced *qrf* expression, *frq^10^frqccg-2*. Strains were grown in light, transferred to darkness, samples collected at the indicated times, DNA was isolated and digested with *Sau*3AI (S) or *Dpn*II (D) and processed for Southern hybridizations using a PLRE specific probe. WT DNA isolated from dark grown cultures was included as a control to compare differences in migration of methylated DNA. (D) To obtain a side-by-side comparison of the methylation pattern for the mutant strains used in this study, DNA obtained from the DD16 time point was taken for each strain and treated as in panel A, B and C. (E) The level of DNA methylation in the samples from (D) was quantified by performing densitometry on the region indicated in (D) and plotted versus the molecular weight marker. It is clear from this figure that *Δchd1* is hypermethylated whereas *frq^9^*, *dsfrh*, and *frq^10^frqccg-2* are all hypomethylated relative to WT.

In considering common elements among the genes whose methylation is influenced by CHD1, it became clear that all are also regulated by light and by the components of the circadian clock. To examine this in more detail we followed DNA methylation in clock-defective strains (Figure 6, Figure S6). *frq^9^* is a loss-of-function allele in which a frame-shift mutation results in premature termination; therefore, *frq^9^* encodes a protein that is incapable of establishing circadian negative feedback [22]. Cultures containing *frq^9^* were grown at 25°C in constant light and then transferred to darkness, and DNA was isolated and digested using the restriction endonucleases *Sau*3AI and *Dpn*II followed by Southern blot analysis. We found a marked reduction of the slower migrating DNA in the *Sau*3AI digest compared with WT, indicative of hypomethylated DNA in *frq^9^* (Figure 6A, compare lanes 1 and 2 with lanes 3–24). We next assayed how loss of FRH affected DNA methylation by using a strain that expresses an inducible hairpin RNA (*dsfrh*) that causes silencing of the *frh* gene [12]. Under conditions in which FRH expression was reduced, we found a noticeable decrease in DNA methylation at *frq* that was similar to that in the *frq^9^* strain (Figure 6B). We conclude that normal levels of methylation require proper expression of core clock genes or at least FRQ-FRH dependent negative feedback, suggesting that both FRQ and FRH play an important, although perhaps indirect, role in regulating methylation at *frq*.

An antisense *frq* transcript is also expressed from the *frq* locus [41]. Because antisense transcripts are known to be involved in X-chromosome inactivation (*Xist/Tsix*) [48], and because RNA-dependent DNA methylation (RdDM) requires formation of double stranded RNAs [49], we sought to examine the role of the *frq* antisense transcript in establishment of normal methylation. To test this, we used a strain *frq^10^frqccg-2* [41] in which the 3′ region of *frq*, containing the promoter of this antisense transcript *qrf*, is replaced with the 3′ region of *eas* (*ccg-2*). This drastically reduces expression of the antisense transcript and results in a small phase lag in the oscillator [41]. In *frq^10^frqccg-2*, methylation is still present but is significantly reduced compared to WT (Figure 6C).

To further quantify the level of DNA methylation in WT and to allow direct comparison among *Δchd1* and the clock mutant strains, we examined methylation at a single time point, DD16 (Figure 6D) and then performed a densitometric analysis on the methylated region (Figure 6E). We confirmed that the observed differences were not a result of the *ras-1^bd^* mutation that is present in *frq^9^*, *dsfrh*, and *frq^10^frqccg-2*, but not in WT or *Δchd1*. Because there was no significant difference in the methylation patterns of WT and *ras-1^bd^*, we conclude that *ras-1^bd^* has no apparent effect on the promoter methylation at *frq* (Figure S6B and S6C). The degree of methylation enhancement in *Δchd1* and reduction in the strains bearing mutations in core clock components compared to WT is apparent. In the time course experiments, we routinely observed a reduction in the overall amount of methylated DNA at later time points (DD36 and beyond), which may suggest that the level of DNA methylation is reduced after prolonged incubations in the absence of light/dark cycles.

### *frq* DNA is methylated by DIM-2

**Figure 7:**
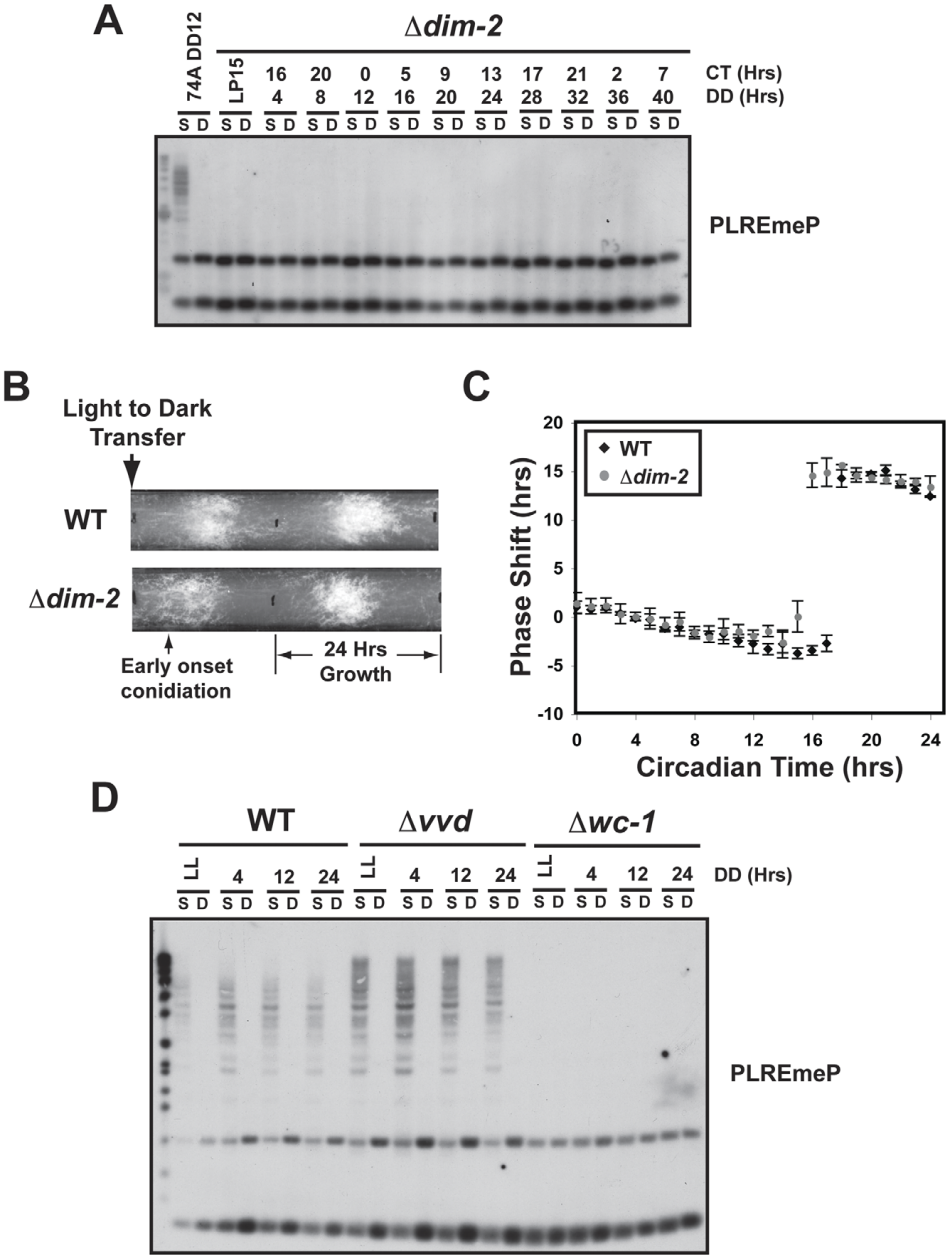
DIM-2 is required for *frq* methylation and effects the phase of the clock. (A) DNA was isolated from a *Δdim-2* strain and digested with *Sau*3AI (S) or *Dpn*II (D) and probed with the PLRE probe. (B) Phenotype of *Δdim-2* compared to WT grown on race tubes. Data are presented for two circadian cycles. (C) A phase-response curve comparing WT and *Δdim-2* over one full circadian cycle. (D) DNA methylation analysis of *Δvvd* and *Δwc-1* compared to WT grown under a limited circadian time course.

To better understand the possible role of DNA methylation in circadian regulated gene expression, we identified the methyltransferase responsible for this activity. Consistent with prior studies showing the DNA methyltransferase (DNMT) DIM-2 (defective in methylation) to be required for all observed DNA methylation in *Neurospora* [50], we found that *Δdim-2* lacked all detectable methylation at *frq* (Figure 7A). This allowed us to examine the role of DNA methylation in circadian rhythmicity.

Circadian clock-regulated conidiation is still observed in *Δdim-2*, *ras-1^bd^* strains using race tubes, indicating that DNA methylation in not essential for rhythmicity per se (Figure 7B, Figure S7). There was, however, a slight phase-advance of approximately 2 hours in strains lacking DIM-2 (Figure 7B, Figure S7A). To confirm the phase-advance, we generated a Phase Response Curve (PRC) comparing *Δdim-2* to WT (Figure 7C). Normally, a pulse of light will advance or delay the phase of the clock depending on the time when the pulse of light is given. In WT, the greatest shift in phase occurs around CT18; however, in *Δdim-2* the largest shift was observed at CT15, further supporting the 2 hour phase-advance. A representative set of race tubes from the CT16 time point is shown in Figure S7B to highlight the phase shift difference between WT and *Δdim-2*. These results suggest that DNA methylation plays an ancillary but supportive role in clock regulation; namely, overt rhythms are not affected yet the onset of circadian clock-regulated expression is affected.

Further supporting the correlation between altered DNA methylation and altered circadian phase, we found that the *vvd* deletion strain, with its well-documented defects in clock phasing, also has an altered DNA methylation profile (Figure 7D). We note that the *frq* promoter is hypermethylated in *Δvvd*, further supporting the notion that normal DNA methylation is required for proper phasing. We also note that there is no DNA methylation in the *Δwc-1* strain, consistent with a connection between normal expression of the *frq/qrf* locus and normal DNA methylation. The early onset of conidiation observed in *Δdim-2* is consistent with the notion that DNA methylation is involved in transferring regulatory signals to pertinent clock genes thereby delaying the start of clock gene expression.

## Discussion

We set out to understand how chromatin is remodeled at *frq*, and to investigate how this might help establish permissive and non-permissive states for rhythmic transcription. Our work led us to discover a link between CHD1-catalyzed chromatin remodeling, DNA methylation, and phase determination. We demonstrate that CHD1 contributes to apparent changes in chromatin structure at *frq* and is needed for normal *frq* expression. Surprisingly, we discovered DNA methylation at *frq*, providing the first example of promoter methylation in *Neurospora*, and we found that *frq* and other loci are hypermethylated in *Δchd1* strains. We showed that DIM-2 is the DNMT responsible for methylating *frq* and that deletion of *dim-2* results in a small phase defect.

Changes in DNA methylation at *frq* were also observed in *Δvvd*, and *frq^10^frqccg-2*, and both these strains have phase defects, providing a link between DNA methylation and phasing of clock gene expression. The apparent minor phase advance of approximately two hours in *Δdim-2* is significant considering the circadian timescale. Under normal circadian conditions, the onset of *frq* expression occurs approximately 8 hours after the transition to dark conditions; therefore, a 2-hour advance represents a 25% shift. Moreover, there is probably a FRQ-induced refractory period after the transition to dark conditions where the WCC is inactive due to negative feedback caused by light-expressed FRQ-FRH. Thus WC-mediated transcription cannot occur until light-expressed FRQ is cleared by the proteasome, which takes roughly 4–6 hours. Based on expression profiles of *frq* in *frq^9^* [22] and *frh^10^* [51], we know expression of *frq* is suppressed upon transition to dark with a WT core-oscillator. Thus, under conditions where the negative elements are unaffected, the maximum observable phase advance should be approximately two hours, as found in the *Δdim-2* strain.

In this report, we present data indicating that proper expression of both *frq* and its antisense counterpart, *qrf*, are essential for normal DNA methylation in this region. In fact, WC-1 dependent expression of *frq*, and its antisense noncoding counterpart *qrf*, clearly influences methylation. Interestingly, non-coding RNAs affect the methylation status of corresponding genes in other systems (i.e. mammals, plants) [52], [53], raising the possibility that the novel methylation that we report also depends on RNA. Further work is needed to explore this possibility but it is noteworthy that the mammalian clock gene *mPer2* has promoter methylation [54], [55]. The hypermethylation phenotype observed in *Δchd1* may result from improper expression of the sense/antisense pair or spurious transcripts, which would ultimately lead to defects in disiRNA (Dicer-independent small interfering RNA) production. A recent report indicates that in *Neurospora* sense/antisense transcripts produce disiRNA [56] and these may direct the DNA methylation through an unknown mechanism. Perhaps such RNAs could play a role in DNA methylation. An unrelated possibility is that CHD1 is involved in RNA processing of the *qrf* and/or 3′ end of the *frq* transcripts and this is coupled to a chromatin remodeling event whose misregulation leads in some way to the observed hypermethylation phenotype. A role for CHD1 in mRNA processing has been described in mammalian cell culture [57].

It is clear that CHD1 is needed for proper *frq* expression and does appear to remodel chromatin at *frq*. However, the role CHD1 plays in clock-regulated expression remains complicated by the residual remodeling that occurs in the absence of CHD1, and additional work is also needed to fully understand how DNA methylation influences *frq* expression. Regardless of the eventual role discovered for promoter methylation though, the simple fact that transient, apparently facultative, and presumably regulated DNA methylation occurs in the fungal kingdom is noteworthy. That this phenomenon went undiscovered for so long, but is so apparent in cultures grown at room temperature in light dark cycles, may serve to highlight the value of studying organisms under conditions that approximate the conditions in which they live in nature.

## Materials and Methods

### Strains and growth conditions

The wild-type (WT) Oak Ridge strain FGSC2489 (74A) strain or the clock WT strain, 328-4 (*ras-1^bd^*, *A*) were used as controls and parent strains for crosses. Strains generated in this study are shown in Table S1. The *Δchd1* strain was generated by the knockout consortium [40], obtained from the Fungal Genetics Stock Center (FGSC, University of Missouri, Kansas City), and crossed to 74A generating XB99-1. Ascospores were germinated on complete media and genotyped by Southern blot for the hygromycin resistance cassette (Figure S1). The *Δdim-2*, *ras-1^bd^* strain, XB98-3, was generated by crossing FGSC8594 to 328-4 and spores were selected for hygromycin resistance and then screened on race tubes for the presence of the *ras-1^bd^* allele. For luciferase strains, the plasmid pVG110 [42], containing the full-length *frq* promoter fused to luciferase, was first transformed into *rid^−^* strains, FGSC9014 and FGSC9015, and targeted to the *his-3* locus (Larrondo et al., in preparation). XB100-9 (*Δchd1*::*hph*, *rid^−^*, *A*) was crossed to FGSC9015 containing pVG110 and spores were selected on hygromycin, then screened for luminescence to obtain XB105-13. Growth media on race tubes consisted of 1X Vogel's salts, 0.1% glucose, 0.17% arginine [58]. Luciferase readings were measure using 12.5 µM luciferin as described [42].

The method used to generate the phase-response curve is outlined elsewhere [47]. Briefly, the phase response curve was generated using standard race tubes grown in the light for 12 or 24 hours and then transferred to dark conditions. After the third day of growth, individual race tubes were subjected to a 15 minute light pulse at the indicated circadian time and then placed back into a dark incubator for an additional 5–6 days (8 days of total growth). The phase on day 1 and on day 5 was determined using Chrono software and plotted as the difference of the two days.

The liquid culture assays were performed in media (2% LCM) containing 1X Vogel's salts, 0.17% arginine with 2% glucose and grown at 25°C. Conidia were used to seed mycelial mats in 75 mm Petri dishes and 0.5 cm plugs were cut from these and grown in 100 ml cultures. Mats were harvested by filtration, frozen in liquid nitrogen and ground in a mortar and pestle. Time course experiments have been described previously [22].

### ChIP and chromatin-remodeling assays

The ChIP assays and oligonucleotides were identical to those previously described for WC-2 [18]. For MNaseI assays, nuclei were isolated in the dark as previously described [10], [13] and then subjected to limited MNaseI digestion following established protocols [59]. Equal amounts of nuclei were resuspended in MNaseI buffer A, treated with 1.0 ml 0.1 M CaCl_2_, incubated at 37°C for 1.5 minutes, and then varying amounts of MNaseI were added and the nuclei incubated for an additional 1.5 minutes. The enzyme was inactivated by the addition of Proteinase K (100 µg/ml) in TENS buffer (20 mM Tris-HCl, pH 7.4, 200 mM NaCl, 2 mM EDTA, 2% SDS), and the nuclei were incubated for a minimum of 2 hours to remove the chromatin. The DNA was purified by phenol chloroform extraction-EtOH precipitation and digested with *Nco*I as the secondary enzyme. DNA fragments were resolved on a 1.5% agarose gel, transferred to positively charged nitrocellulose, and probed with *frqP4* specific probe. Remodeling at the antisense promoter was performed using the identical protocol except *Eco*RI was used as the restriction enzyme with *frqP18*. MeDIP-chip experiments were performed as previously described [45].

### Northern, Southern, and Western blot

Standard Northern and Southern blots were performed using digoxigenin label DNA probes following Roche guidelines. All of the oligonucleotides used for probes are contain in Table S2. RNA was isolated from cells using a hot-phenol extraction or Trizol and 15 or 25 ug/ul of RNA were fractionated on a 1.3% agarose formaldehyde gel [22]. Quantitative RT-PCR was performed as described [60]. DNA was isolated using the Puragene Kit following the manufactures' protocol. Immunoblot analysis was as described [19].

## Supporting Information

Figure S1 Circadian, growth, and molecular characterization of the *Δchd1* strain. (A) WT 74A (FGSC 2489), *ras-1^bd^* (328-4) and *Δchd1* were grown on race tubes. In addition to the genetic interaction with *ras-1^bd^*, the *Δchd1* strain has an overall retarded growth phenotype. Confirmation of *chd1* deletion strain by Southern blot using probes for *chd1* (B) and *hph* (C). (D) Schematic of *chd1* locus showing the relative locations of the probes.
(PDF)

Figure S2 CHD1 is required for normal WT circadian rhythms Expression of a *frq-luc* reporter construct was measured on race tubes in WT and *Δchd1*. (A) An actual race tube showing the areas used to obtain recordings. The luminescence was recorded for WT and *Δchd1* isolates and plotted for the whole tube (B and C) and inoculation point (D and E). (F) Schematic representation of the sectional analysis used to monitor rhythms at the growth front. Light emitted from the area marked by each section was measured for the entire span of days required for strains to grow down the length of the tube. Bioluminescence levels are and plotted for WT (G) and *Δchd1* (H). Each separate line traces the amount of light emitted by the culture within each small section. In G it is clear that cultures in each section remain rhythmic whereas in H, the *Δchd1* strain shows no rhythm.
(PDF)

Figure S3 Analysis of remodeling in the *frq* promoter. We consistently observed subtle remodeling in the region surrounding the PLRE and TSS in a WT strain and this remodeling differed in *Δchd1*. (A) Schematic representation of the *frq* promoter highlighting the region around the PLRE and TSS. (B and D) Partial MNase I digestion probing the region around the PLRE and TSS. (C and E) Quantification of the remodeling observed in WT compared to *Δchd1*. The light-dependent remodeling that occurs at the TSS (Site 3) appears to be CHD1 independent. However, there is a slight defect in CHD1-dependent remodeling at the PLRE in the dark (Sites 1 and 2). Under normal WT conditions, *frq* is expressed in early subjective morning DD12 and the relative amount of site 1 is roughly half that of site 2 that we surmise is indicative of a more open chromatin state. At DD24 (and DD4, Panel D and E) in WT, a time when *frq* transcription is minimal, we see two predominant bands in the PLRE region, which are roughly at a 50∶50 ratio (site 1 and site 2) indicating a movement of nucleosomes to a more closed condensed state. In *Δchd1* there does not appear to be a relative increase in Site 1 at the at the DD24 (or DD4, Panel D and E) time point and there is no discernable difference in Site 1 intensity relative to Site 2 between the DD12 and DD24 time points indicating a lack of chromatin remodeling at this site. Admittedly, this is difficult to observe because there is only a subtle change observed in this MNaseI assay, but the important point is that the relative intensity of site 1 to site 2 in *Δchd1* is always lower compared to WT where the relative ratios of site 1 to site 2 are roughly equal at times when *frq* expression is low (DD4 and DD24); These data are further compounded by the large proportion of undigested DNA from *Δchd1* nuclei (see methylation analysis). Yet, this experiment is very reproducible as the independent biological replicates show similar results.
(PDF)

Figure S4 A representative methylation Southern blot was probed for *frq* promoter DNA (PLREmeP) and then stripped and re-probed for a region of the mitochondrial genome (mitoP). WT DNA from two different time points (DD12 and DD16) were digested with *Sau*3AI and *Dpn*II, resolved on a 1.0% agarose gel, transferred to nitrocellulose and probed as described in Material and Methods. To ensure complete cutting with *Sau*3A1, a 2X concentration is shown next to a standard amount.
(PDF)

Figure S5 DNA Methylation at other loci. WT and *Δchd1* methylation southern blots were stripped and reprobed for the *frq* coding region (A and B), the *ccg-2* promoter (C and D), the *vvd* promoter (E and F) and the ψ_63_ region (G and H).
(PDF)

Figure S6 DNA methylation at *wc-1*. (A) Data obtained from the MeDIP Chip experiment indicated that in addition to *frq*, *wc-1* was also methylated in *Δchd1*. This was confirmed by a methylation sensitive Southern Blot using *BfuCI* (an isoschizomer of *Sau3AI*) and *DpnII*. (B and C) A side-by-side comparision was done to examine if there were any methylation difference in *ras-1^bd^* compared to an isogenic WT. It is clear there are no significant differences other than variations due to loading.
(PDF)

Figure S7 The *Δdim-2* strain that has no DNA methylation retains a functional circadian clock but displays an altered phase. (A) Three independent isolates from a cross containing both *Δdim-2* and *ras-1^bd^* were grown on race tubes. The phase on day 1 for each strain is shown at the right. (B) A sample racetube used to generate the phase response curve is shown. Note the large difference in phase shift on Day 6 between WT and *Δdim-2*.
(PDF)

Table S1 Strains used in this study.
(PDF)

Table S2 Oligonucleotides.
(PDF)
