# Supplementary figures and images for: CHD1 Remodels Chromatin and Influences Transient DNA Methylation at the Clock Gene frequency
Source: PLoS Genet. 2011 Jul 21;7(7):e1002166. doi: 10.1371/journal.pgen.1002166 (PMC3140994; doi:10.1371/journal.pgen.1002166)

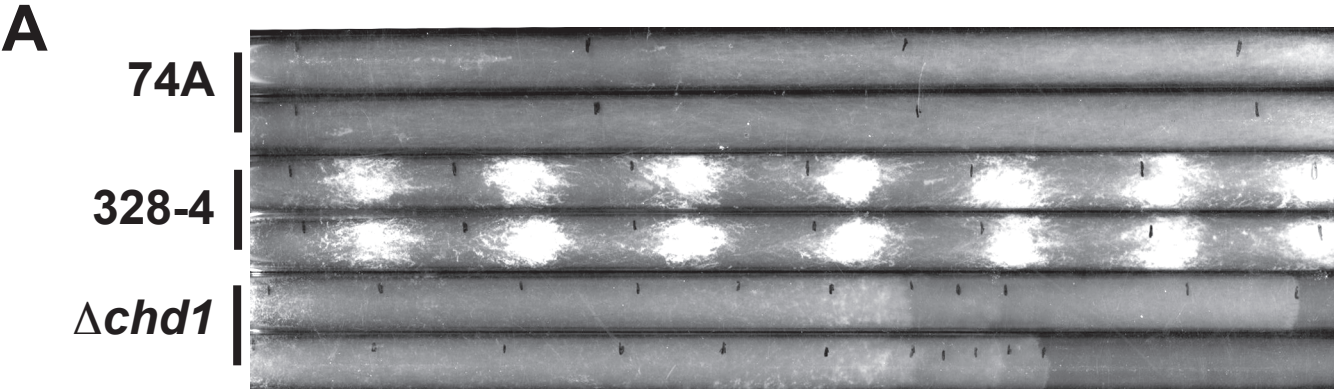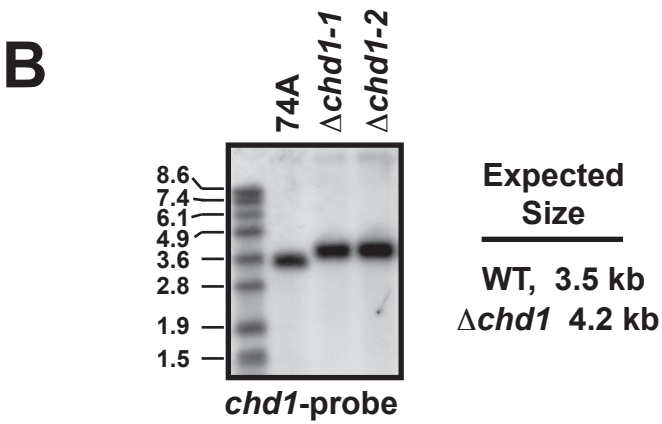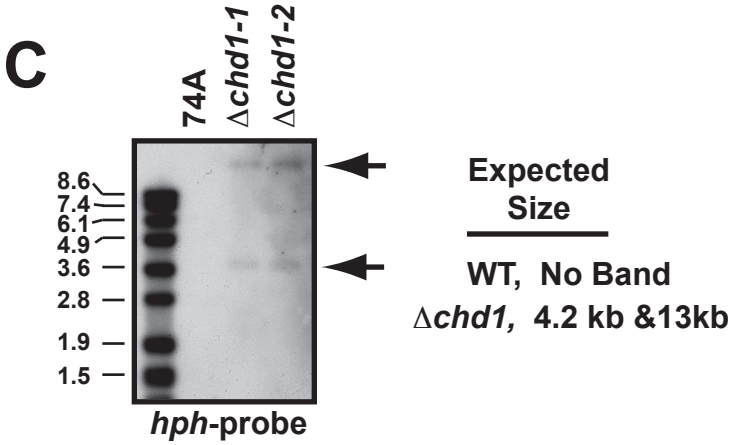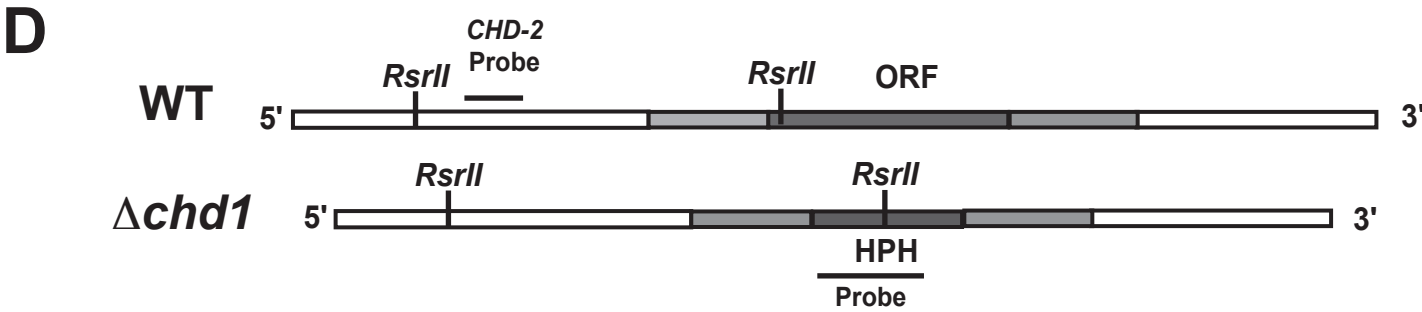

Supplement: Figure S1 — Circadian, growth, and molecular characterization of the Δchd1 strain. (A) WT 74A (FGSC 2489), ras-1bd (328-4) and Δchd1 were grown on race tubes. In addition to the genetic interaction with ras-1bd, the Δchd1 strain has an overall retarded growth phenotype. Confirmation of chd1 deletion strain by Southern blot using probes for chd1 (B) and hph (C). (D) Schematic of chd1 locus showing the relative locations of the probes. (PDF) [file pgen.1002166.s001.pdf]

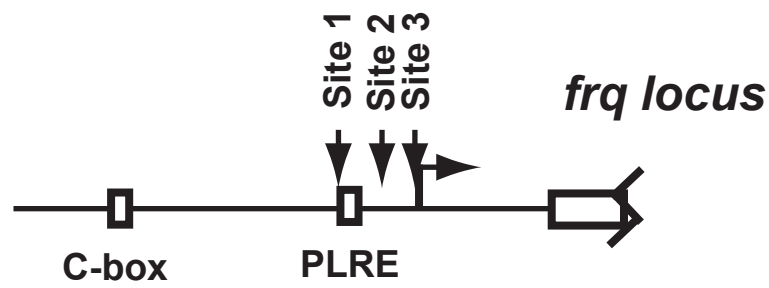**B**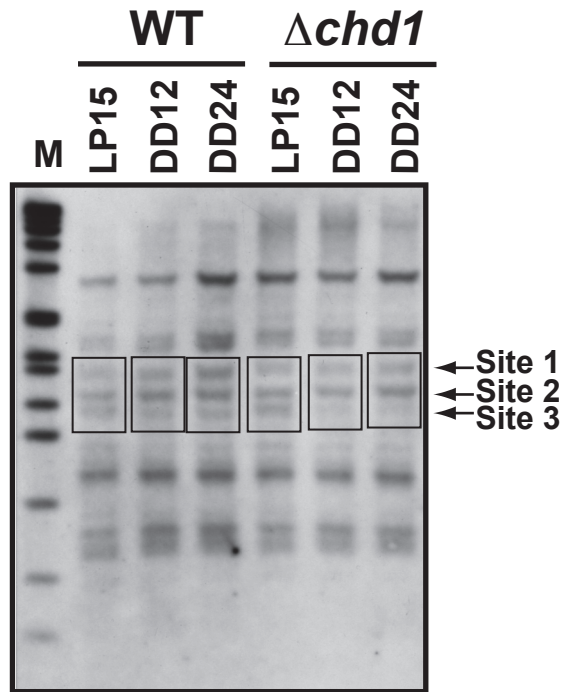**C**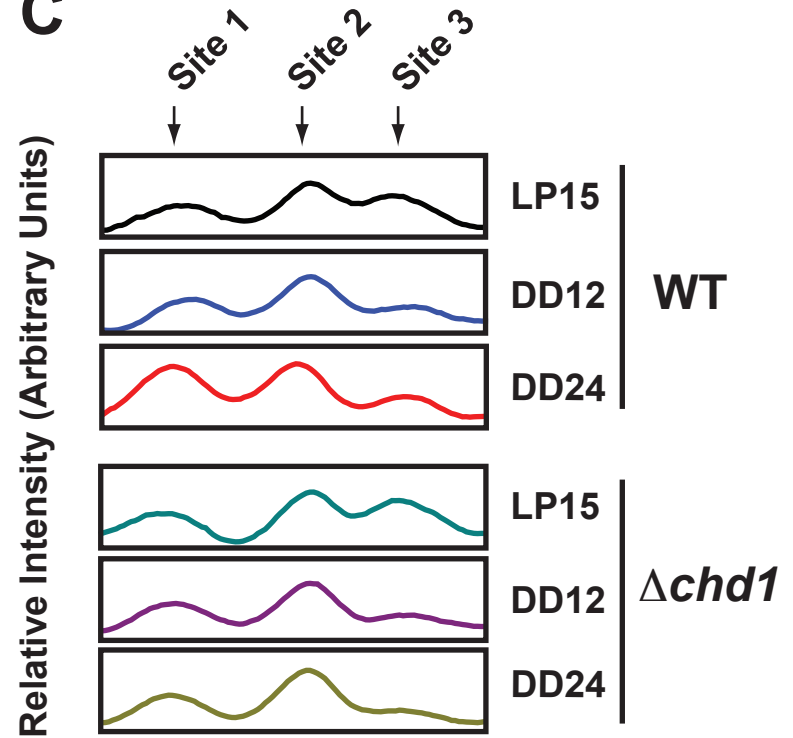**D**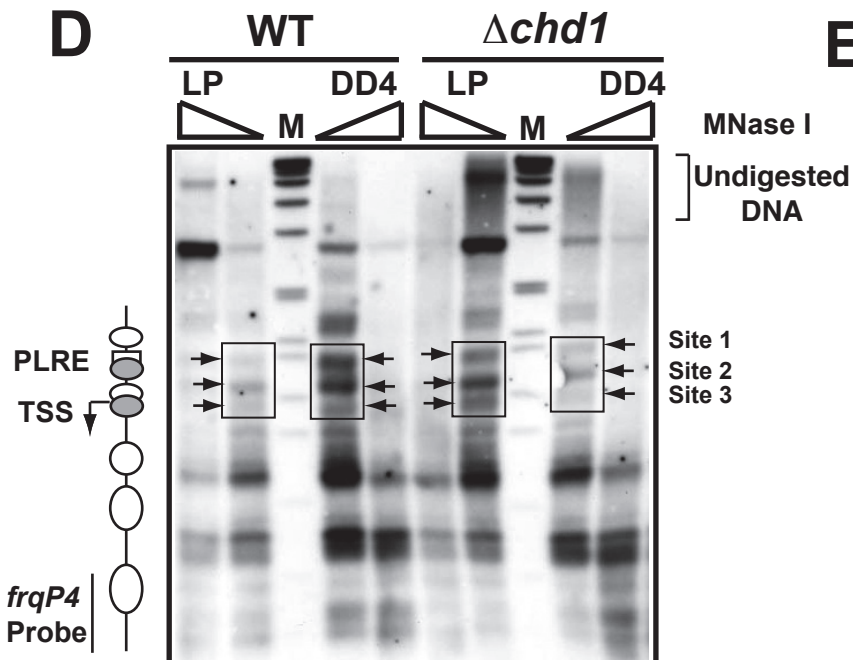**E**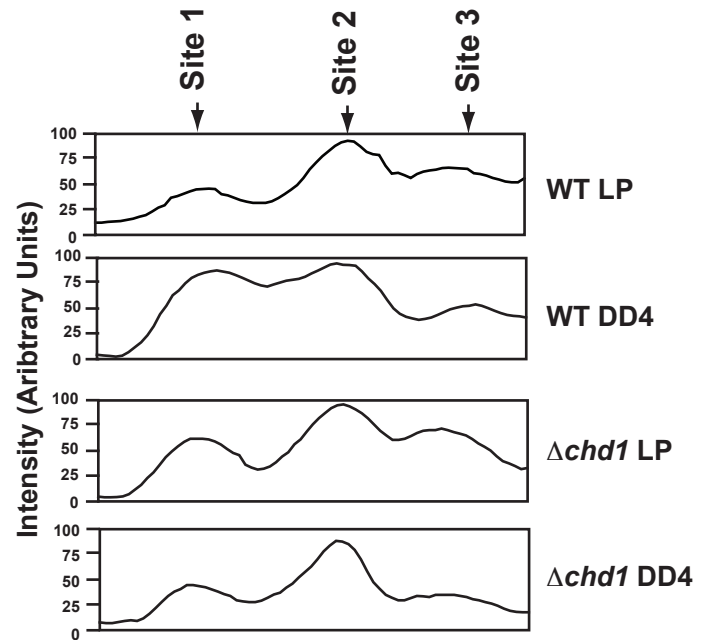

Supplement: Figure S3 — Analysis of remodeling in the frq promoter. We consistently observed subtle remodeling in the region surrounding the PLRE and TSS in a WT strain and this remodeling differed in Δchd1. (A) Schematic representation of the frq promoter highlighting the region around the PLRE and TSS. (B and D) Partial MNase I digestion probing the region around the PLRE and TSS. (C and E) Quantification of the remodeling observed in WT compared to Δchd1. The light-dependent remodeling that occurs at the TSS (Site 3) appears to be CHD1 independent. However, there is a slight defect in CHD1-dependent remodeling at the PLRE in the dark (Sites 1 and 2). Under normal WT conditions, frq is expressed in early subjective morning DD12 and the relative amount of site 1 is roughly half that of site 2 that we surmise is indicative of a more open chromatin state. At DD24 (and DD4, Panel D and E) in WT, a time when frq transcription is minimal, we see two predominant bands in the PLRE region, which are roughly at a 50∶50 ratio (site 1 and site 2) indicating a movement of nucleosomes to a more closed condensed state. In Δchd1 there does not appear to be a relative increase in Site 1 at the at the DD24 (or DD4, Panel D and E) time point and there is no discernable difference in Site 1 intensity relative to Site 2 between the DD12 and DD24 time points indicating a lack of chromatin remodeling at this site. Admittedly, this is difficult to observe because there is only a subtle change observed in this MNaseI assay, but the important point is that the relative intensity of site 1 to site 2 in Δchd1 is always lower compared to WT where the relative ratios of site 1 to site 2 are roughly equal at times when frq expression is low (DD4 and DD24); These data are further compounded by the large proportion of undigested DNA from Δchd1 nuclei (see methylation analysis). Yet, this experiment is very reproducible as the independent biological replicates show similar results. (PDF) [file pgen.1002166.s003.pdf]

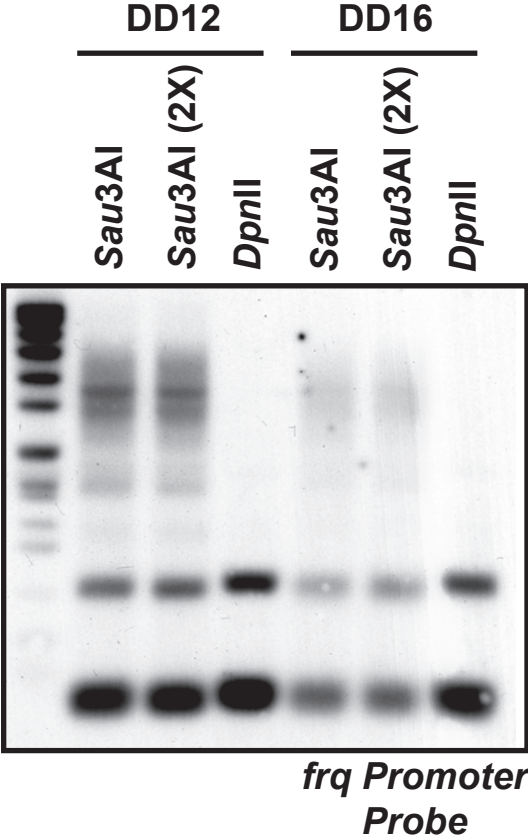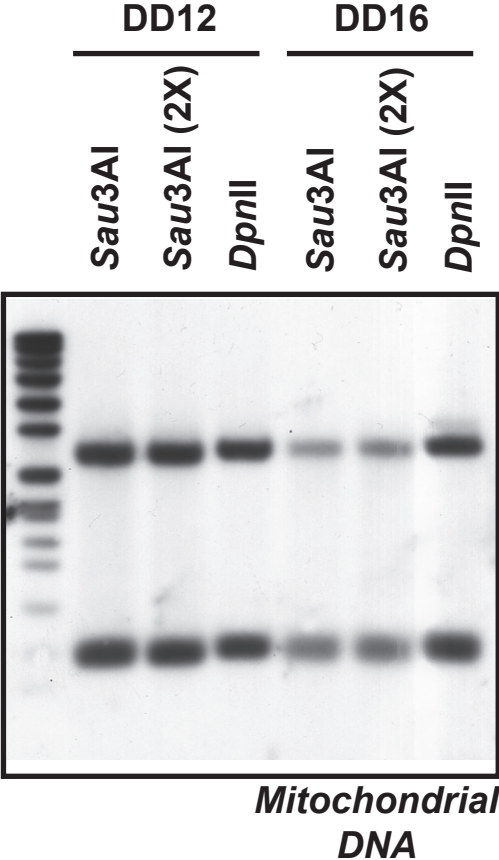

Supplement: Figure S4 — A representative methylation Southern blot was probed for frq promoter DNA (PLREmeP) and then stripped and re-probed for a region of the mitochondrial genome (mitoP). WT DNA from two different time points (DD12 and DD16) were digested with Sau3AI and DpnII, resolved on a 1.0% agarose gel, transferred to nitrocellulose and probed as described in Material and Methods. To ensure complete cutting with Sau3A1, a 2X concentration is shown next to a standard amount. (PDF) [file pgen.1002166.s004.pdf]

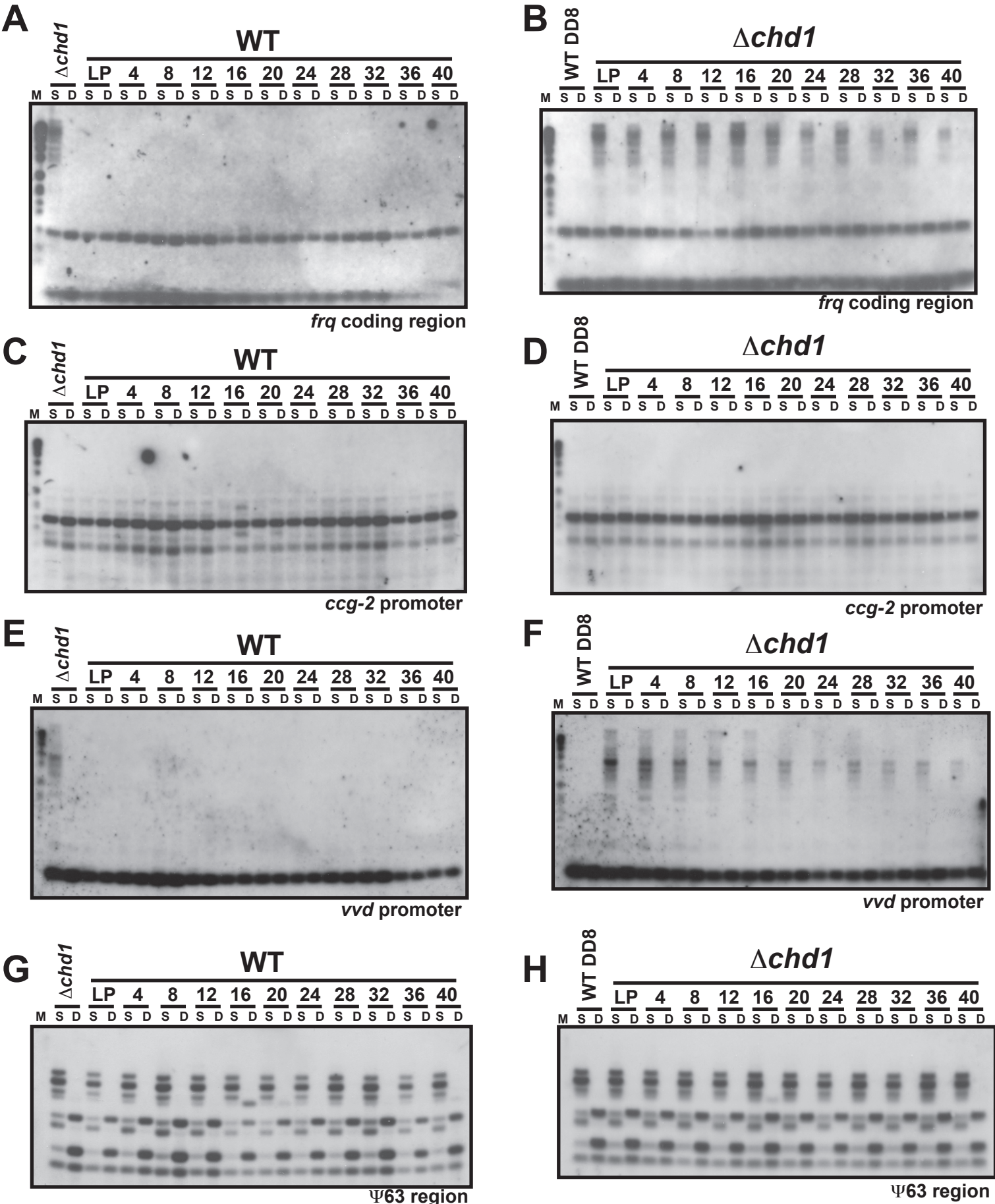

Supplement: Figure S5 — DNA Methylation at other loci. WT and Δchd1 methylation southern blots were stripped and reprobed for the frq coding region (A and B), the ccg-2 promoter (C and D), the vvd promoter (E and F) and the ψ63 region (G and H). (PDF) [file pgen.1002166.s005.pdf]

A

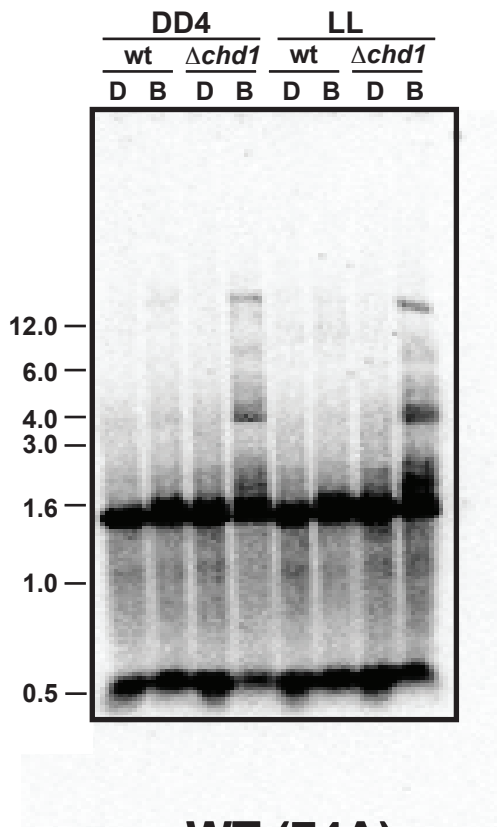

B

WT (74A)

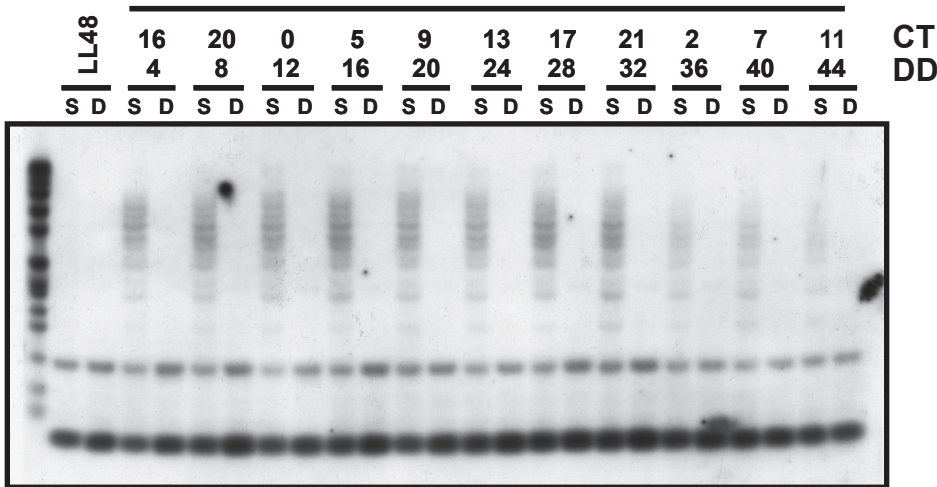

C

*ras-1*<sup>bd</sup>

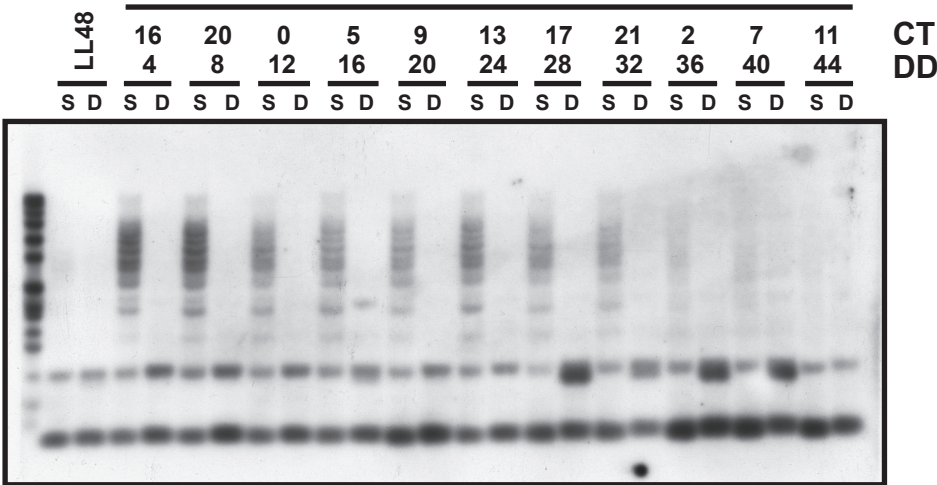

Supplement: Figure S6 — DNA methylation at wc-1. (A) Data obtained from the MeDIP Chip experiment indicated that in addition to frq, wc-1 was also methylated in Δchd1. This was confirmed by a methylation sensitive Southern Blot using BfuCI (an isoschizomer of Sau3AI) and DpnII. (B and C) A side-by-side comparision was done to examine if there were any methylation difference in ras-1bd compared to an isogenic WT. It is clear there are no significant differences other than variations due to loading. (PDF) [file pgen.1002166.s006.pdf]

**A**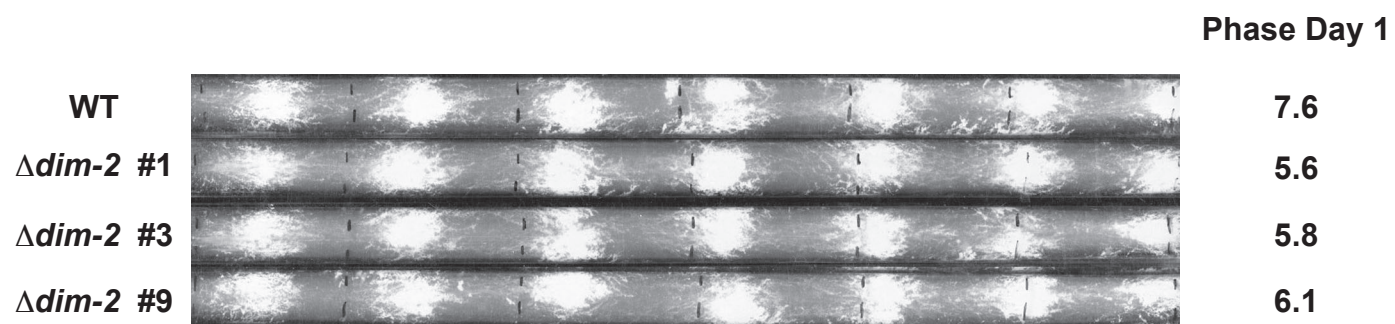**B**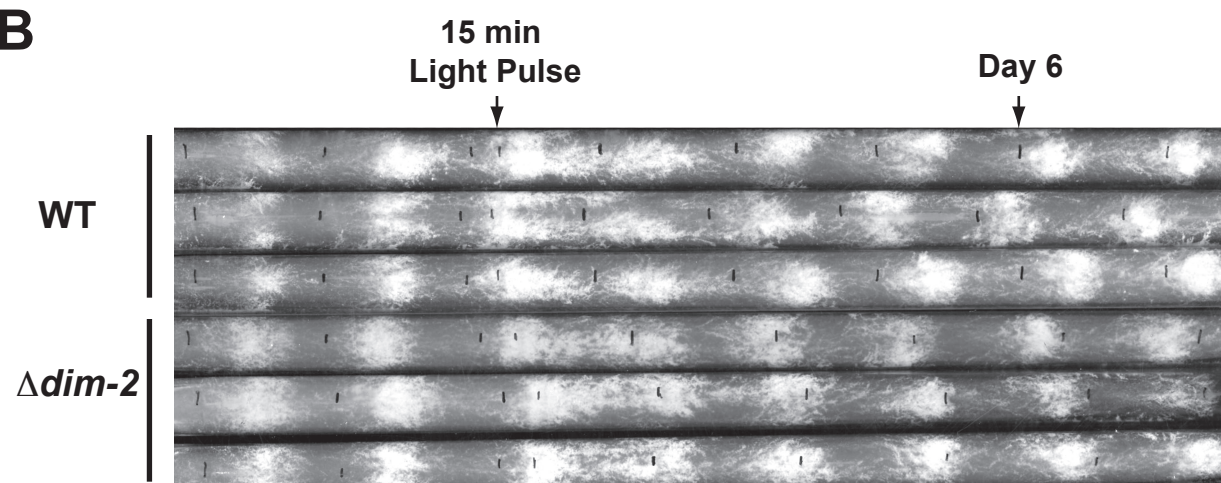

Supplement: Figure S7 — The Δdim-2 strain that has no DNA methylation retains a functional circadian clock but displays an altered phase. (A) Three independent isolates from a cross containing both Δdim-2 and ras-1bd were grown on race tubes. The phase on day 1 for each strain is shown at the right. (B) A sample racetube used to generate the phase response curve is shown. Note the large difference in phase shift on Day 6 between WT and Δdim-2. (PDF) [file pgen.1002166.s007.pdf]
