# Supplementary material for: CHD1 Remodels Chromatin and Influences Transient DNA Methylation at the Clock Gene frequency
Source: PLoS Genet. 2011 Jul 21;7(7):e1002166. doi: 10.1371/journal.pgen.1002166 (PMC3140994; doi:10.1371/journal.pgen.1002166)

Supplemental Figure 2

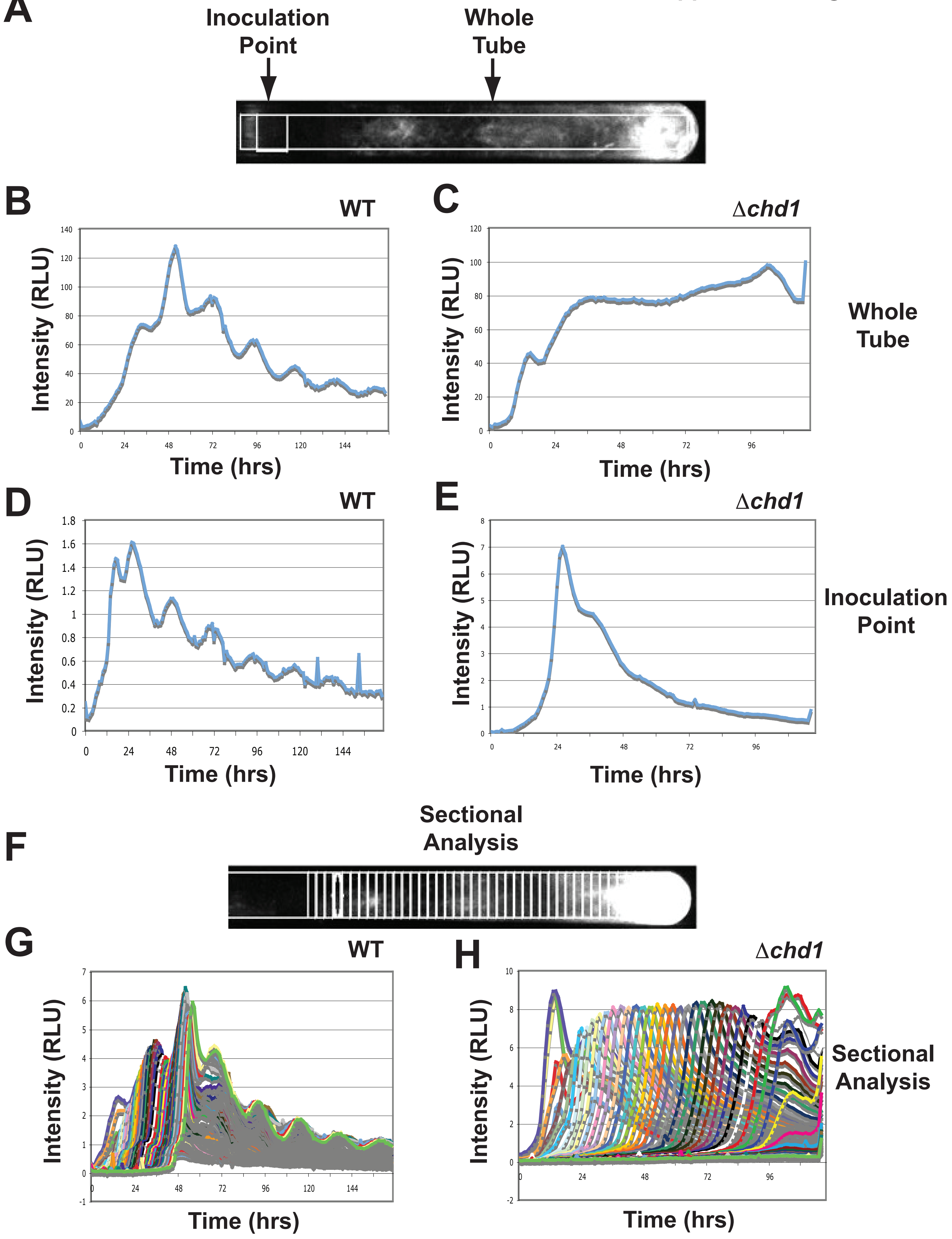

Supplement: Figure S2 — CHD1 is required for normal WT circadian rhythms Expression of a frq-luc reporter construct was measured on race tubes in WT and Δchd1. (A) An actual race tube showing the areas used to obtain recordings. The luminescence was recorded for WT and Δchd1 isolates and plotted for the whole tube (B and C) and inoculation point (D and E). (F) Schematic representation of the sectional analysis used to monitor rhythms at the growth front. Light emitted from the area marked by each section was measured for the entire span of days required for strains to grow down the length of the tube. Bioluminescence levels are and plotted for WT (G) and Δchd1 (H). Each separate line traces the amount of light emitted by the culture within each small section. In G it is clear that cultures in each section remain rhythmic whereas in H, the Δchd1 strain shows no rhythm. (PDF) [file pgen.1002166.s002.pdf]
