# Supplementary material for: CHD1 Remodels Chromatin and Influences Transient DNA Methylation at the Clock Gene frequency
Source: PLoS Genet. 2011 Jul 21;7(7):e1002166. doi: 10.1371/journal.pgen.1002166 (PMC3140994; doi:10.1371/journal.pgen.1002166)
Supplement: Table S1 — Strains used in this study. (PDF) [file pgen.1002166.s008.pdf]

Table S1

Strains Used in this Study

Strain	Genotype	Reference
FGSC 2489	74-OR23-1V A (WT)	FGSC
328-4	A, *ras-1*^{bd}	Froehlich et al., 2002
94-40	*A, ras-1*^{bd} *frq*^{9}	Loros et al., 1986
frq^{10}*, frqccg-2*	*A, ras-1*^{bd}*, frq*^{10}*, his-3*$^{+}$::*pfrqccg-2*	Kramer et al., 2003
FGSC8594	*a, his-3, dim-2::hph*	FGSC
FGSC14805	*a, NCU03060::hph, mus52::Bar*	Colot et al., 2006
FGSC9014	*A, rid*$^{-}$*, his-3*$^{-}$	FGSC
FGSC9015	*a, rid*$^{-}$*, his-3*$^{-}$	FGSC
FGSC9014 (pVG110)	*A, rid*$^{-}$*, his-3*$^{+}$*::pVG110*	Lorrondo et al.
FGSC9015 (pVG110)	*a, rid*$^{-}$*, his-3*$^{+}$*::pVG110*	Lorrondo et al.
XG-1	*a, mus52::hph, ras-1*^{bd}	Lorrondo et al.
XB99-1 (Δchd1)	*a, NCU03060::hph*	This Study
XB98-3	*a, dim-2::hph, ras-1*^{bd}	This Study
XB100-9	*A, rid*$^{-}$*, NCU03060::hph*	This Study
XB105-13	*A, rid-, NCU03060::hph, his-3*$^{+}$*::pVG110*	This Study
