# Supplementary material for: CHD1 Remodels Chromatin and Influences Transient DNA Methylation at the Clock Gene frequency
Source: PLoS Genet. 2011 Jul 21;7(7):e1002166. doi: 10.1371/journal.pgen.1002166 (PMC3140994; doi:10.1371/journal.pgen.1002166)
Supplement: Table S2 — Oligonucleotides. (PDF) [file pgen.1002166.s009.pdf]

Table S2

Oligonucleotides

frqP4F	TGGACCCCAGGAAAGCCCAG
frqP4R	GAAGAAGCACGCTAGAACGG
frqP18F	CATCGCCGAACGCTTGGCAG
frqP18R	GACGTCCTCCATCGAACTAC
CBMeP1F	CACTTGCTGCCCTTCATCAG
CBMeP2R	CCTACATATTGCATTTGGCG
PLREMeP1F	CCTCAGCATTTTGTCGTGAG
PLREMeP1R	GGATCGAAGTGTGTTTGCGG
MitoP2F	ggaaatacattacctacac
MitoP2R	ctagtcctttatcttctgg
